# Mechanochemically Carboxylated Multilayer Graphene for Carbon/ABS Composites with Improved Thermal Conductivity

**DOI:** 10.3390/polym10101088

**Published:** 2018-10-01

**Authors:** Laura Burk, Matthias Gliem, Fabian Lais, Fabian Nutz, Markus Retsch, Rolf Mülhaupt

**Affiliations:** 1 Freiburg Materials Research Center (FMF), Stefan-Meier-Straße 21, D-79104 Freiburg, Germany; laura.burk@fmf.uni-freiburg.de (L.B.); matthias.gliem@fmf.uni-freiburg.de (M.G.); fabianlais@web.de (F.L.); 2 Institute for Macromolecular Chemistry of the Albert-Ludwigs-University Freiburg, Stefan-Meier-Straße 31, D-79104 Freiburg, Germany; 3 Department of Chemistry, University of Bayreuth, Universitätsstraße 30, D-95447 Bayreuth, Germany; fabian.nutz@uni-bayreuth.de (F.N.); markus.retsch@uni-bayreuth.de (M.R.)

**Keywords:** mechanochemistry, graphene, nanocomposite, thermal conductivity, ABS

## Abstract

Dry ball milling of graphite under carbon dioxide pressure affords multilayer-functionalized graphene (MFG) with carboxylic groups as nanofiller for composites of carbon and acrylonitrile–butadiene–styrene copolymers (ABSs). Produced in a single-step process without requiring purification, MFG nanoplatelets are uniformly dispersed in ABS even in the absence of compatibilizers. As compared to few-layer graphene oxide, much larger amounts of MFG are tolerated in ABS melt processing. Unparalleled by other carbon nanofillers and non-functionalized micronized graphite, the addition of 15 wt % MFG simultaneously results in a Young’s modulus of 2550 MPa (+68%), a thermal conductivity of 0.321 W∙m^−1^∙K^−1^ (+200%), and a heat distortion temperature of 99 °C (+9%) with respect to neat ABS, without encountering massive embrittlement and melt-viscosity build-up typical of few-layer graphene oxide. With carbon filler at 5 wt %, the Young’s modulus increases with increasing aspect ratio of the carbon filler and is superior to spherical hydroxyl-functionalized MFG, which forms large agglomerates. Both MFG and micronized graphite hold promise for designing carbon/ABS compounds with improved thermal management in lightweight engineering applications.

## 1. Introduction

The quest for efficient thermal management in lightweight engineering prompts challenges for the development of engineering plastics exhibiting improved thermal conductivity. Since most engineering polymers are thermal insulators they are rendered thermally conductive by incorporating highly thermally conductive fillers such as metal powders, aluminum oxide, boron nitride, graphite or by carbon fiber reinforcement [1]. However, the large amounts of micron-sized thermally conductive fillers required for improving thermal conductivity frequently impair mechanical properties. Opposite to micron-sized fillers nanofillers with an average diameter below 100 nm have much larger specific surface areas and considerably lower percolation threshold. Moreover, the uniform dispersion of an extremely large number thermally conductive nanofillers enables interfacial engineering and converts bulk polymers into interfacial polymers exhibiting new property profiles.

Among conducting nanofillers carbon nanotubes are most widely studied with respect to the influence of their sizes, shapes, modifications, dispersion, and interfaces on thermal conductivity and mechanical properties of the corresponding polymer nanocomposites [2]. In recent years, graphene as a 2D carbon polymer with honeycomb-like array of sp^2^-hybridized carbon atoms have emerged as potential nanofillers exhibiting exceptional combination of high stiffness (Young’s modulus of 1 TPa), barrier resistance against gas and fluid permeation, and enormous electrical (6000 S∙cm^−1^) and thermal conductivity (5000 W∙m^−1^∙K^−1^) [2,3,4,5]. Today the wide range of graphene applications includes electrode materials, lubricants, and polymer nanocomposites [6,7]. Following the pioneering advances of by Geim and Novoselov in 2004, several bottom-up and top-down synthetic routes have emerged for producing graphene [8]. In top-down syntheses, which is economically more favorable with respect to nanofiller production, graphite is exfoliated to produce either defect-free or functionalized few- and multi-layer graphene [9]. Coleman’s solvent-mediated shear exfoliation of graphite in solvents with matched polarity such as *N*-methyl-pyrrolidone affords defect-free single-layer graphene dispersions with graphene content up to 1 g∙L^−1^ [10,11]. Stable aqueous dispersions of up to 2 g∙L^−1^ graphene oxide nanoplatelets are readily obtained by means of underwater plasma exfoliation of graphite [12]. Electrochemical graphite exfoliation is achieved in electrolytes such as inorganic acids and salts or ionic liquids [13,14,15,16]. In sulfuric acid as electrolyte dispersions of functionalized two-layer graphene with a lateral size of 30 µm are obtained [15]. The reduction of graphene oxide in halide salt melts produces graphene sheets [17]. However, most of these top-down processes, especially those for preparing defect-free graphene, fail to produce cost-competitive easy-to-disperse thermally conductive carbon nanofillers useful in engineering plastic applications [18]. Opposite to defect-free graphene, functionalized few- and multi-layer graphene is readily derived from graphite and tailored for carbon/polymer composite applications [19,20]. Typically, in multi-step processes, graphite is intercalated with sulfuric acid, oxidized, and then chemically or thermally reduced to produce graphene oxide nanofillers [21,22,23]. The resulting graphene oxide bears hydroxyl, phenol, and carboxylic acid groups [24]. Owing to the presence of functional groups and structural defects such as pores graphene oxide has a lower modulus and lower electrical and thermal conductivities as compared to defect-free graphene [25,26]. In contrast, comminution of graphite yields micronized graphene as well as non-functionalized graphene nanoplatelets (GnPs) consisting of less than 10 graphene layers [27]. Dry ball milling of graphite in a planetary ball mill in the presence of dry ice, red phosphorous or gases like nitrogen, hydrogen, and sulfur dioxide affords selectively edge-functionalized graphene (MFG) in a single-step solventless process [27,28,29,30,31]. The energy of impact and friction is sufficient to break carbon bonds and the resulting highly reactive carbon species react with air, carbon dioxide, and even with inert gases such as nitrogen. Opposite to the two-step formation of thermally reduced graphite oxide (TRGO), the mechanochemical functionalization combined with graphite exfoliation in a single process step does not require either the use of explosive graphite oxide as intermediate or laborious filtration and purification processes.

The presence of functional groups is essential for dispersing few-layer graphene oxide as well as MFG in various polymers ranging from polypropylene to thermoplastic polyurethanes and acrylonitrile–butadiene–styrene copolymers (ABSs) [32,33,34]. ABSs represent an engineering thermoplastic exhibiting excellent processability by injection molding, attractive stiffness/toughness balance, chemical resistance, and dimensional stability [35,36,37]. Both ABS and low-temperature impact resistant ABS blends with polycarbonate serve as matrix polymers of nanocomposites [36]. Nanofiller dispersion is achieved through several processes such as melt compounding, solvent-mediated blending, and coagulation of dispersion blends prior to melt processing and through solvent-based casting processes used for preparing thin ABS films [34,35,38]. Melt processing of ABS spans from injection molding and extrusion to additive manufacturing by means of fused deposition modeling [34,39]. Graphite, micronized graphite, GnPs, and multi-walled carbon nanotubes have been applied as carbon fillers for ABS [34,35,36,39,40]. Cardonna et al. reported on the thermal behavior of GnPs/ABS nanocomposites revealing strong impact of the lateral particle size on thermal conductivity. By adding GnPs the thermal conductivity was enhanced by up to +79% [34]. According to Jan and coworkers the dispersion of few-layer graphene in ABS films accounted for improved matrix reinforcement as evidenced by a tensile strength increase up to +43% as compared to +22% for multi-walled carbon nanotubes. Functionalized graphene is readily dispersed within the SAN matrix [35]. Similar results were reported by Gao et al. who dispersed few-layer graphene oxide in ABS by means of their solvent-based coagulation process [38]. Pour and coworkers improved the Young’s modulus of PC/ABS blends by +30% upon incorporating 3.5 wt % GnPs [36]. 

Whereas most studies focus on mechanical, thermal and morphological properties of ABS compounds with GnPs, micron-sized graphite, TRGO and other carbon nanofillers, little is known with respect to the influence carbon filler types, sizes, shapes and functionality on the thermal conductivity of carbon/ABS nanocomposites. To the best of our knowledge, to date no MFG/ABS composites are disclosed in the open literature. Herein we report on the preparation and characterization of the thermal, mechanical and morphological properties of MFG/ABS compounds which are compared with carbon/ABS benchmark compounds containing commercial carbon fillers like micronized graphite (G-1.5 µm), functionalized graphene (C-750) derived from intercalated graphite, and graphene oxide (TRGO) obtained by thermal reduction of graphite oxide at 750 °C. Special emphasis is placed upon examining the influence of carbon filler type, size, aspect ratio and functionality on the balance of thermal conductivity and mechanical properties of carbon/ABS compounds.

## 2. Experimental Section

### 2.1. Materials 

Graphite (KFL 99.5) and micronized graphite (G-1.5 µm–V-HF 99.9) were received from AMG Mining (former Kropfmühl AG, Passau, Germany). G-1.5 µm was highly hygroscopic as seen in the elemental analysis and was dried before each usage. C-750 (Grade C 99.0) was purchased from XG Sciences (Lansing, MI, USA). Acrylonitrile–butadiene–styrene copolymers (ABSs) (containing 50% of butadiene, Novodur^®^ Preco P60P50, MFI = 7.35 g∙10 min^−1^ at 220 °C and 10 kg) were obtained from INEOS Styrolution (Frankfurt, Germany). Irganox^®^1010/Irgafos^®^168 (1:1 wt:wt, p.a.) was purchased from BASF (Basel, Switzerland). Isopropyl alcohol (p.a.) was received from Sigma Aldrich (Taufkirchen, Germany). Carbon dioxide (N45) was supplied by Air Liquide (Kornwestheim, Germany).

### 2.2. Production of Carbon-Based Nanofillers

Mechanochemically edge-functionalized graphene was prepared by grinding graphite (Graphite-KFL, 18.6 g) under carbon dioxide pressure (13 bar) in a planetary ball mill (PM 100 from Retsch, Haan, Germany). The ceramic milling chamber (500 mL) made of Y_0.05_Zr_0.95_O_2_ containing ceramic balls (100 balls, *d* = 10 mm made of Y_0.05_Zr_0.95_O_2_) and graphite (18.6 g) was evacuated and then pressurized with carbon dioxide. Milling was performed for 24 h at 250 rpm. 

Thermally reduced graphite oxide (TRGO) was produced in a two-step reaction closely following procedures reported elsewhere in more detail [23]. Typically after the oxidation of graphite (KFL) according to the method reported by Hummers and Offemann the resulting graphite oxide was thermally reduced at 750 °C in a nitrogen atmosphere.

### 2.3. Preparation of Carbon/ABS Nanocomposites

ABS nanocomposites were prepared by melt compounding in a twin-screw mini extruder followed by injection molding. To facilitate a homogeneous filler distribution, the fillers were first coated onto ABS powder to enhance dispersion during subsequent melt processing. Typically, carbon filler dispersions were prepared by sonicating them in isopropyl alcohol (5 g∙L^−1^) using the Sonopuls HD 3200 from Bandelin (Berlin, Germany) (KE76-sonotrode, 40 min pulsed, 40% amplitude). After adding processing stabilizers (0.1 wt % Irganox^®^1010/Irgafos^®^168 1:1) the resulting carbon dispersion was mixed with ABS powder. Isopropyl alcohol was then evaporated and the solid was dried in an oil pump vacuum. Processing of the ABS nanocomposites was performed using a co-rotating twin screw XploreTM micro compounder (XploreTM from DSM, Geleen, Netherlands, 5.5 mL, 260 °C, 1 min, 120 rpm) followed by injection molding (T_transfer_ 260 °C, T_mold_ 80 °C, p (max)_injection_ 9 bar).

### 2.4. Instrumental Analysis

Elemental analysis (EA) was performed with a VarioEl device from Elementar Analysensysteme GmbH (Langenselbold, Germany) to determine the content of carbon, hydrogen, and nitrogen by combustion analysis. Therefore, the sample was with WO_3_ as a combustion catalyst enriched and combusted under oxygen, followed by gas chromatographic analysis. The oxygen content was identified by electron dispersive X-ray scattering using a Quanta 250 FEG from FEI (Hillsboro, OR, USA) with an INCAx-act-add-on from Oxford Instruments (Abingdon, UK) and an acceleration voltage of 20 kV.

Electrical conductivity of the fillers was measured with a four-point measurement arrangement. For that purpose, a thin film of the filler (*d* = 41 mm) was deposited by vacuum filtration on a polyester-based membrane with an average pore size of 0.2 µm from Pieper Filter GmbH (Bad Zwischenahn, Germany). The electrical conductivity σ was obtained by the inverse specific sheet resistance under consideration of a correction factor with respect to the spherical sample geometry. The composite materials’ electrical conductivity was determined by a two-point measurement arrangement. The rectangularly shaped test specimens (15 × 6 × 2 mm^3^) were contacted with conductive silver purchases from Kemo Electronic (Geestland, Germany), and the resulting resistivity was measured with a Keithly electrometer 617 from Keithley (Cleveland, UH, USA). The calculation of the electrical conductivity *σ* is based on the measured value of the resistance multiplied by the quotient of the distance between the silver contacts and the cross section vertical to the direction of the measuring direction (Equation (1)).

(1) $$\sigma =\;\frac{1}{R}\cdot \frac{l}{A}.$$

Fourier transform infrared spectroscopy (FTIR) of the carbon-based fillers was detected with a Vektor 22 from Bruker (Billerica, MA, USA) using KBr tablets. Each spectrum was recorded by 32 scans with a resolution of 2 cm^−1^ and an additional background and baseline correction.

Morphology’s characterization was done by both transmission electron microscopy (TEM) and scanning electron microscopy (SEM). TEM was performed on a Zeiss LEO 912 from Omega (Oberkochen, Germany), which was operated with an acceleration voltage of 120 kV. The sample preparation of the fillers occurred by deposition on a copper grid from dispersion in THF (3 mg∙mL^−1^, 15 min sonication). The preparation of ultrathin nanocomposite sections (~200 nm) was carried out with a Leica Ultracut UCT microtome from Leica Microsystems GmbH (Wetzlar, Germany) at −120 °C.

Specific surface area was characterized by measuring the nitrogen adsorption according to Brunauer–Emmett–Teller analysis (BET). The measurements were conducted on a Soptomatic 1990 from Porotec (Hofheim, Germany).

Mechanical characterization was done by both tensile testing and impact strength examining five test specimens per sample. Tensile testing was conducted in accordance to DIN ISO 527-1/2_5A using a Zwick Z-005 from ZwickRoell (Ulm, Germany). Every test specimen was aligned vertically in a 2.5 kN grip with a clamping length of 50 mm. Test speed was 50 mm min^−1^, and the tension was detected by a 5 kN load sensor. Notched Izod impact strength was investigated under standard conditions according to DIN EN ISO 180 using a Zwick Pendelum (1 J) from ZwickRoell (Ulm, Germany).

Determination of the melt flow index (MFI) was done according to the testing standard of DIN EN ISO 1133-1 using a MI-4 from Göttfert (Buchen, Germany).

Thermal conductivity was obtained according to Equation (2):

(2) $$\kappa \left(T\right)=\;\alpha \left(T\right)\cdot c_{p}\left(T\right)\cdot \rho \left(T\right).$$

Thermal diffusivity α was determined by laser flash analysis on an XFA 500 XenonFlash apparatus from Linseis (Selb, Germany) operated with an InSb infrared detector. Prior to the measurements the samples’ thickness was determined using a Litematic VL-50 from Mitutoyo (Neuss, Germany). Additionally, the samples were coated with a thin layer (≤15 µm) of graphite on each side. The temperature-dependent specific heat capacity c_p_ was measured by differential scanning calorimetry (DSC) calibrated with a sapphire standard. Density *ρ* was measured using a buoyancy balance.

Thermal properties were investigated by DSC, dynamic mechanical analysis (DMA) and thermogravimetric analysis (TGA). DSC measurements were performed on DSC 204 F1 Phoenix from Netzsch (Selb, Germany). Glass transition temperature (*T*_g_) was explored within the second heating cycle owing a heating rate of 10 K∙min^−1^ within a temperature range of −150–260 °C. DMA was conducted with a Dynamic Mechanical Analyzer Q800 and a single cantilever clamp from TA Instruments (New Castle, DE, USA) on a rectangular test specimen. Applied frequency and strain were 1.0 Hz and a strain of 0.1%, respectively. The temperature range was set between −140 and 160 °C with a ramp of 3 K∙min^−1^. The maximum of tanδ was used for determination of *T*_g_. TGA was carried out on a Thermobalance STA 409 from Netzsch (Selb, Germany). The measurement was done between 50 and 650 °C under nitrogen using a heating rate of 10 K∙min^−1^ and a nitrogen flow of 75 cm^3^∙min.

The heat distortion temperature (HDT) Vicat B50 was measured according to DIN EN ISO 306 with a Öko-Vicat Tester from Coesfeld (Dortmund, Germany).

## 3. Results and Discussion

### 3.1. Carbon Fillers Derived from Graphite

**Figure 1:**
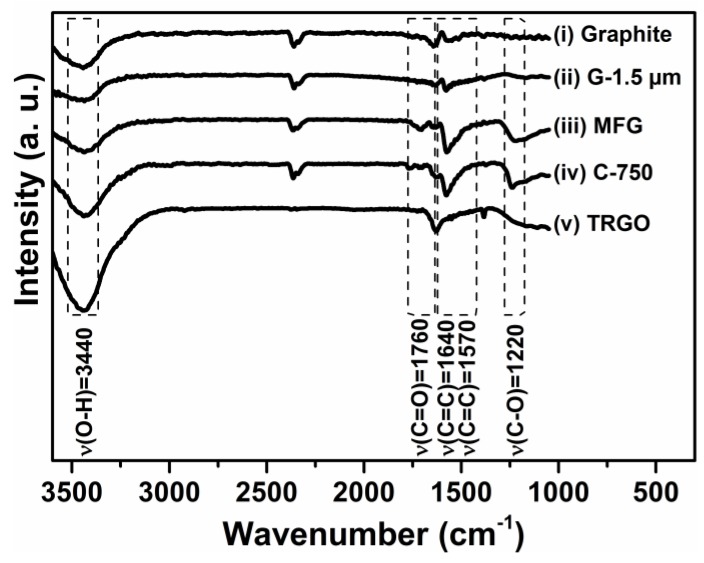
FTIR spectra of graphite (i), micronized graphite G-1.5 µm (ii), mechanochemically functionalized multilayer graphene MFG (iii), commercial functionalized graphene C-750 (iv), and thermally reduced graphite oxide TRGO (v), which was prepared by thermal reduction of graphite oxide at 750 °C.

**Figure 2:**
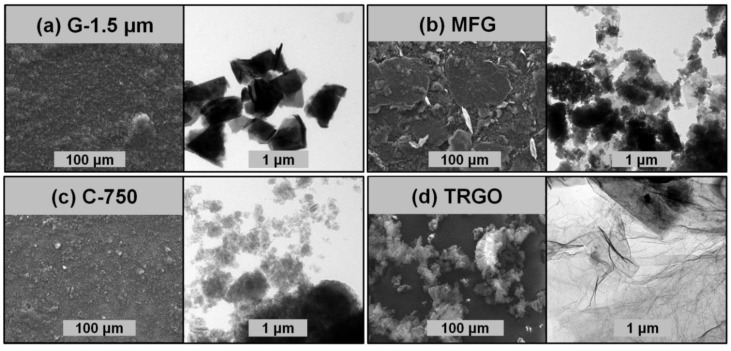
Morphology, as determined by SEM (1st and 3rd column) and TEM (2nd and 4th column), of G-1.5 µm (**a**), MFG (**b**), C-750 (**c**), and TRGO (**d**).

Particle size, shape, number of graphene layers, and functionalization of carbon fillers derived from graphite was varied over a wide range. The properties of the graphite-based carbon fillers are listed in Table 1. Figure 1 shows IR spectra of mechanochemically carboxylated multilayer-functionalized graphene (MFG), graphene oxide (TRGO) produced by thermal reduction of graphite oxide at 750 °C, functionalized graphene (C-750 from XG-Sciences) derived from graphite intercalates and non-functionalized micronized graphite (G-1.5 µm from AMG Mining) with an average flake size of 1.5 µm. The corresponding particle morphologies are displayed in Figure 2. Similar to procedures reported previously, edge-selective carboxylated MFG was prepared by dry grinding graphite under a carbon dioxide pressure of 13 bar [41]. The use of mill chambers and balls made of abrasion-resistant zirconia ceramics eliminated the massive metal abrasion typical of steel mill tools, which require laborious purification by acid treatment in a second step. High shear forces, ball impact, and the interplay of centrifugal and Coriolis forces during milling in a planetary ball mill accounted for the delamination of graphite intercalated with carbon dioxide and the formation of highly reactive carbon species which react with carbon dioxide predominantly at the edges of the resulting graphene nanoplatelets. This is in accord with previous reports by Jeon et al. who obtained selectively edge-carboxylated MFG by dry ball milling graphite in the presence of dry ice [30]. As is apparent from Table 1, MFG has a significantly higher oxygen content of 8.7 wt % as compared to 1.2 wt % for graphite and 5.4% for micronized graphite. In accord with previous reports in the literature, only the IR spectra of MFG (see Figure 1) showed the carbonyl vibration band at 1710 cm^−1^ and the C–O vibration band at 1220 cm^−1^ typical for the presence of carboxylic acid groups [42]. Preferably the mechanochemical carboxylation by milling graphite under carbon dioxide pressure required a milling duration of 24 h. As compared to graphite, which consists of a very large number of graphene layers, milling under carbon dioxide pressure drastically reduced the particle size and produced a mixture of GnPs with broad particle size distribution. In contrast, TRGO produced by thermal reduction of graphite oxide at 750 °C contained mainly hydroxyl and phenol groups together with a minute amount of carboxyl groups (see trace iv in Figure 1), since carboxyl and epoxy groups of graphite oxide thermally decompose during thermolysis at 750 °C [22,23,43]. In fact, the pressure build-up associated with this degradation during thermolysis accounts for graphite exfoliation [23]. Hence, few-layer graphene stacks were formed as evidenced by the microscopic imaging in Figure 2 and the high specific surface area of 520 m^2^∙g^−1^ (see Table 1), which was considerably higher with respect to that of graphite, micronized graphite, and MFG. The formation of few-layer TRGO bearing hydroxyl groups, as verified by the presence of IR absorption bands at 1180 cm^−1^, is in accord with previous reports in the literature [44]. In this family of graphite-derived carbon fillers, TRGO exhibited the highest oxygen content of 15.7 wt %, corresponding mainly to hydroxyl groups [19,20,45]. Unlike the other spherical or platelet-like carbon fillers only TRGO showed the wrinkled structures typical of single- and few-layer graphene. Clearly, TRGO possessed the highest aspect ratio within this series of carbon fillers. Only C-750 exhibited a higher surface area of 770 m^2^∙g^−1^ but a much lower oxygen content of 8.8 wt %, corresponding to the presence of mainly hydroxyl and few carboxylic acid groups as evidenced by IR bands at 1760 and 1220 cm^−1^ (see Figure 1). This is in accordance with the literature [42]. Opposite to MFG, TRGO, and micronized graphite, C-750 exclusively contained spherical nanoparticles but no nanoplatelets. The FTIR spectrum of G-1.5 µm (see Figure 1) revealed only the presence of aromatic C=C bending vibration bands without any indication of functionalization. In accordance with reports in the literature, the IR absorption observed around 3440 cm^−1^ and the oxygen content of G-1.5 µm are attributed to absorbed water [46].

### 3.2. Carbon/ABS Nanocomposites

All carbon fillers were melt-compounded with ABS using a twin-screw mini extruder followed by injection molding. Preferably, to improve filler dispersion, suspensions of the fillers in iso-propanol were coated onto ABS powders prior to melt compounding. At carbon filler loadings of 5 and 15 wt %, the melt flow index (MFI) depended on specific surface areas. As compared to G-1.5 µm and MFG, both C-750 and TRGO exhibited significantly large specific surface areas well above 500 m^2^∙g^−1^ accompanied by drastically impaired melt processing as reflected by the massive MFI decay as shown in Table 2. With a filler content of 15 wt %, the MFI of TRGO/ABS and C-750/ABS was well below the detection level. The MFI drastically decreased with increasing aspect ratio within the series TRGO > C-750 > MFG > G-1.5 µm. Opposite to TRGO and C-750, significantly larger amounts of G-1.5 µm and MFG were tolerated in ABS melt processing.

**Figure 3:**
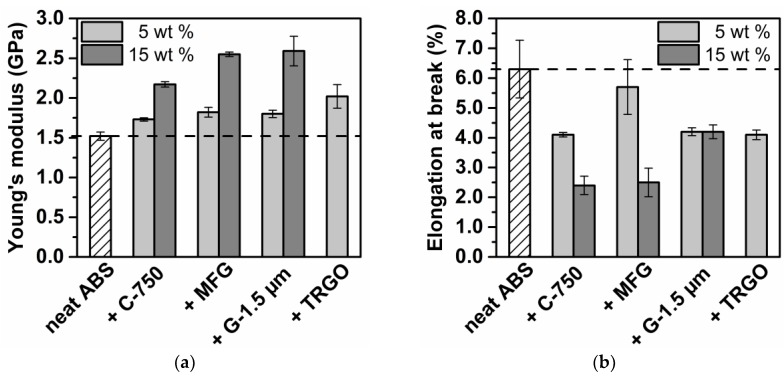

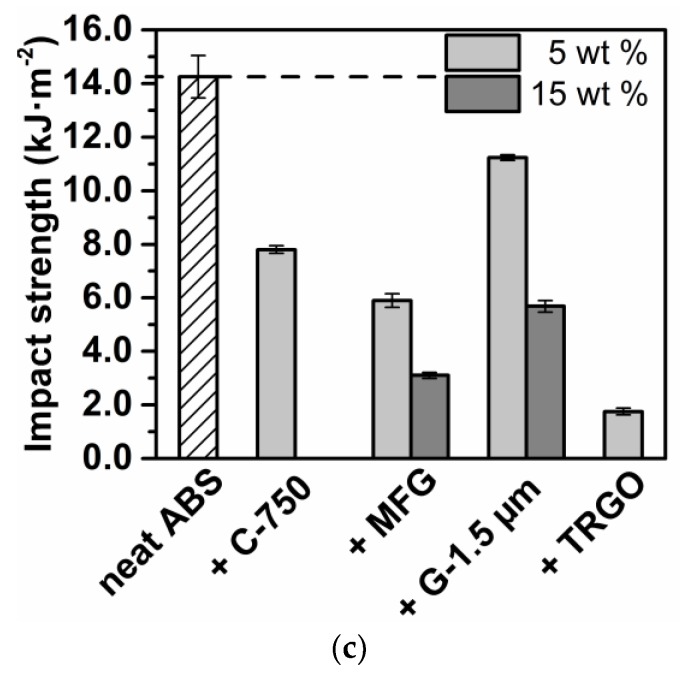
Young’s modulus (**a**), elongation at break (**b**), and impact strength (**c**) of carbon/ABS nanocomposites as a function of carbon type and content.

**Figure 4:**
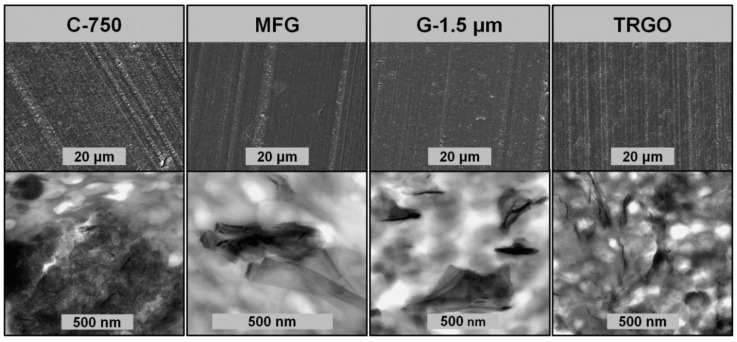
Morphology imaged by means SEM (above) and TEM (below) of carbon/ABS nanocomposites as a function of the filler type at a filler loading of 5 wt %.

The mechanical properties of carbon/ABS nanocomposites were characterized by stress–strain and Izod impact testing. The mechanical properties together with the electrical and thermal conductivities of carbon/ABS compounds as a function of carbon filer type and content are listed in Table 3 and graphically displayed by the bar charts displayed in Figure 3. At both 5 and 15 wt % filler content, the stiffness of carbon/ABS nanocomposites, as expressed by Young’s modulus, increased with increasing aspect ratio and increasing aspect ratio in the series C-750 < MFG < G-1.5 µm < TRGO. At a carbon filler content of 5 wt %, the highest increase (+27%) of Young’s modulus (1920 MPa) with respect to neat ABS (1520 MPa) was achieved by adding TRGO. However, while processing problems restricted the TRGO content to 5 wt %, the Young’s modulus significantly increased at 15 wt % loading to 2550 MPa (+67%) for MFG and to 2740 MPa (+80%) for G-1.5 µm. Although the stiffness enhancement was achieved at the expense of both elongation at break and Izod impact strength, the encountered embrittlement was much lower for the addition of MFG and G-1.5 µm with respect to TRGO and C-750 (see Figure 3). According to microscopic imaging of carbon/ABS by means of SEM and TEM (see Figure 4), all carbon fillers with a high aspect ratio were uniformly dispersed within the ABS matrix. However, only the TRGO addition yielded dispersed few-layer graphene, whereas both G-1.5 µm/ABS and MFG/ABS contained much larger sub-micron GnPs consisting of graphene stacks. In contrast, when processed under identical processing conditions, C-750/ABS exhibited large agglomerates at a filler loading of 5 wt %. According to Differential Scanning Calorimetry (DSC) and Dynamic Mechanical Analysis (DMA), neither filler type nor filler content affected the glass temperatures of the polybutadiene and SAN phases. This finding is in contrast to reports by Gao et al. who proposed graphene accumulation within the SAN attributed to strong π–π interactions between graphene and styrene units [38]. In accordance with observations by Cardonna and Wang, the thermal degradation of carbon/ABS nanocomposites at 400 °C, as determined by thermogravimetric analysis (TGA), was unaffected by both filler type and filler loading [34,37].

The heat distortion temperature (HDT) of carbon/ABS composites was determined as a function of filler type and filler content (see Table 3). With filler loading at 5 wt %, the TRGO/ABS nanocomposite exhibited an HDT of 94 °C, but this increase by 3 °C with respect to neat ABS was achieved at the expense of processability as reflected by the massive MFI decay. The highest HDT of 99 °C was observed for MFG/ABS containing 15 wt % MFG. In contrast to both TRGO and MFG micronized graphite G-1.5 µm failed to improve impact resistance. Most likely the presence of functional groups is essential for improving HDT.

**Figure 5:**
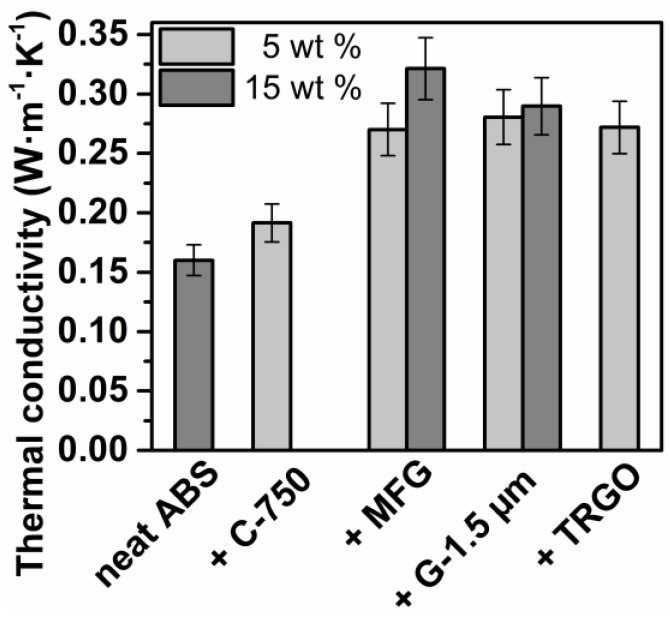
Thermal conductivity of carbon/ABS nanocomposites as a function of the filler type and content.

**Figure 6:**
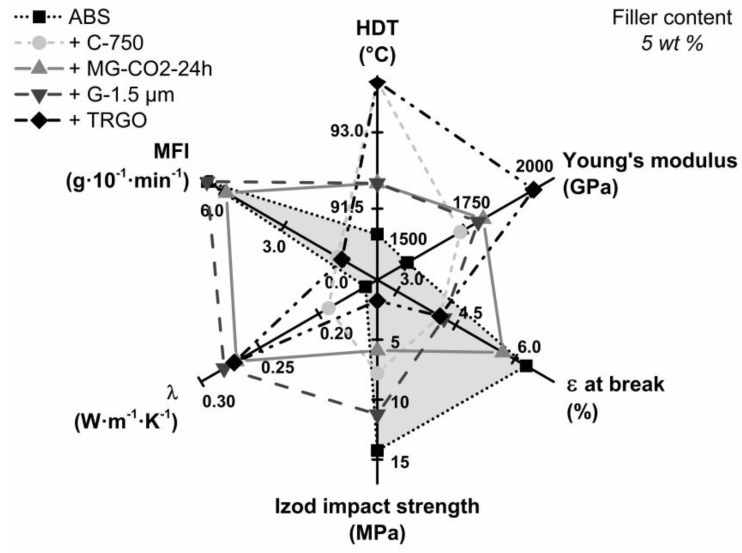
Property profiles of carbon/ABS nanocomposites containing 5 wt % carbon filler as a function of the filler type (gray surface are the properties of neat ABS).

Electrical conductivity of the carbon/ABS nanocomposites was measured by means of a two-point measurement. Only the addition of 5 wt % TRGO rendered TRGO/ABS electrically conductive with rather low electrical conductivity of 2 × 10^−7^ S∙cm^−1^, while all other carbon/ABS composites with filler content values up to 15 wt % were electrical insulators (see Table 3). In view of thermal management of ABS in lightweight engineering, it is desirable to improve thermal conductivity without encountering electrical conductivity [2]. From Table 3 and Figure 5, it is apparent that the addition of all carbon fillers markedly improved thermal conductivity. At 5 wt %, all carbon/ABS composites containing carbon fillers with a high aspect ratio exhibited thermal conductivities around 0.270 W∙m^−1^∙K^−1^ (+170%) with respect to 0.160 W∙m^−1^∙K^−1^ for ABS, whereas the identical amount of spherical C-750 nanoparticles afforded markedly lower thermal conductivity of 0.192 W∙m^−1^∙K^−1^ (+120%). Upon the addition of 15 wt % MFG, thermal conductivity further increased to 0.321 W∙m^−1^∙K^−1^ (+200%), which is somewhat higher with respect to 0.290 W∙m^−1^∙K^−1^ (+ 180%) for G-1.5 µm. The spider diagram displayed in Figure 6 reveals that only MFG simultaneously improves thermal conductivity, heat distortion temperature, and stiffness without drastically impairing melt processability.

## 4. Conclusions

The mechanochemical carboxylation by dry milling graphite in a planetary ball mill under carbon dioxide pressure of 13 bar represents a facile synthetic route to carboxylated multilayer graphene (MFG) serving as multifunctional carbon nanofillers for ABS. Owing to the presence of carboxyl groups, MFG is readily dispersed in ABS melts even in the absence of dispersing aids and compatibilizers. Opposite to conventional functionalized graphene, requiring either multi-step syntheses or expensive processes, MFG is derived from graphite in quantitative yields without requiring solvents or laborious recovery and purification. Particularly the use of ceramic mill containers and balls prevents massive metal abrasion and eliminates laborious and expensive purification by acid treatment typical for steel mill tools. Whereas dispersing functionalized few-layer graphene oxide in ABS is accompanied by massive processing problems owing to drastic build-up of melt viscosity even at low filler content values, much larger amounts of MFG are tolerated in ABS melt processing by injection molding. Unparalleled by other carbon nanofillers and non-functionalized micronized graphite, the addition of 15 wt % MFG simultaneously improves stiffness, as reflected by an increased Young’s modulus of 2550 MPa (+68%), a higher thermal conductivity of 0.321 W∙m^−1^∙K^−1^ (+200%), and a higher heat distortion temperature of 99 °C (+9%) with respect to neat ABS, without encountering massive embrittlement typical of dispersions of thermally reduced graphite oxide. The performance of platelet-like MFG is far superior to functionalized spherical nanoparticles derived from graphite which are much more difficult to disperse and form large agglomerates. Hence, it is imperative during dry ball milling to preserve platelet structure. Like carbon nanotubes the high bulk conductivity of graphene and graphite are not fully transferred to MFG/ABS composites at low filler content. At a carbon filler content of 5 wt %, the Young’s modulus increases with increasing aspect ratio of the carbon filler. Only thermally reduced graphite oxide renders ABS electrically conductive at a content of 5 wt %. MFG and micronized graphite hold great promise for designing carbon/ABS compounds targeting improved thermal management in applications such as power electronics and automotive applications of engineering plastics allocated near electrical engines. In summary, mechanochemistry is a highly promising alternative to producing nanofillers for ABS nanocomposites as well as other polymer hybrid materials with respect to tuning property profiles and simultaneous thermal conductivity, mechanical properties, and dimensional stability without encountering massive processing and cost problems.

## Figures and Tables

**Table 1:** Properties of the carbon fillers.

Sample	C (wt %) ^a^	H (wt %) ^a^	O (wt %) ^b^	*σ* (S∙cm^−1^) ^c^	BET (m^2^∙g^−1^) ^e^
Graphite	99.6	0.2	1.2	3440 ^d^	1
G-1.5 µm	96.7	0.2	5.4	1.2	50
MFG	92.1	0.6	8.7	2.1∙10^−1^	261
C-750	88.8	1.1	8.8	0.3∙10^−1^	770
TRGO	82.4	0.8	15.7	16.0	520

^a^ elemental analysis; ^b^ energy-dispersive X-ray Spectroscopy; ^c^ four-point measurement using a coated filter; ^d^ four-point measurement using a pressed TRGO tablet; ^e^ determined according Brunauer–Emmett–Teller (BET) theory.

**Table 2:** Melt flow index (MFI) of carbon/acrylonitrile–butadiene–styrene copolymer (ABS) nanocomposites ^a^.

Filler Type (wt %)	G-1.5 µm (g∙10^−1^∙min^−1^)	MFG (g∙10^−1^∙min^−1^)	C-750 (g∙10^−1^∙min^−1^)	TRGO (g∙10^−1^∙min^−1^)
5	6.7	5.8	0,4	0.2
15	1.7	0.6	n.d. ^b^	n.d. ^b^

^a^ MFI of neat ABS is 6.7 g∙10^−1^∙min^−1^, ^b^ not detectable owing to severely impaired melt processability.

**Table 3:** Properties of carbon/ABS nanocomposites.

Sample Code	Young’s Modulus ^b^ (MPa)	Elongation at Break ^b^ (%)	Impact Strength ^b^ (kJ∙m^−2^)	Electrical Conductivity (S∙cm^−1^)	Thermal Conductivity ^b^ (W∙m^−1^∙k^−1^)	HDT ^a^ (°C)
ABS	1520 ± 50	6.3 ± 0.8	14.3 ± 0.8	n. d.	0.160 ± 0.013	91
+5% C-750	1720 ± 20	4.1 ± 0.1	7.8 ± 0.1	n. d.	0.192 ± 0.016	94
+15% C-750	2170 ± 40	2.4 ± 0.3	n. d.	n. d.	n. d.	-
+5% MFG	1790 ± 30	5.7 ± 0.6 ^c^	5.9 ± 0.3	n. d.	0.270 ± 0.022	92
+15% MFG	2550 ± 30	2.5 ± 0.3	3.1 ± 0.1	n. d.	0.321 ± 0.026	99
+5% G-1.5 µm	1850 ± 20	4.3 ±.0.1	11.2 ± 0.1	n. d.	0.280 ± 0.023	92
+15% G-1.5 µm	2740 ± 80	4.1 ± 0.2	5.7 ± 0.2	n. d.	0.290 ± 0.024	93
+5% TRGO	1930 ± 20	3.8 ± 0.2	1.8 ± 0.1	2 ∙ 10^−7^	0.272 ± 0.022	94

^a^ heat distortion temperature according to Vicat DIN EN ISO 306 using method B50; ^b^ significance of properties controlled by *t*-tests with the respective sample and ABS as control sample; ^c^ difference to neat ABS is considered to be not statistically significant.
